# Medical viruses: diagnostic techniques

**DOI:** 10.1186/s12985-023-02108-w

**Published:** 2023-07-11

**Authors:** Harsh, Pratima Tripathi

**Affiliations:** Department of Biotechnology, National Institute of Pharmaceutical Education and Research, Bijnor-Sisendi Road, Sarojini Nagar, Near CRPF Base Camp, Lucknow, UP 226002 India

## Abstract

The recent epidemics and pandemics caused by different viruses such as SARS-CoV-2, monkey pox, H1N1, ebola virus etc. have been a cause of mass destruction in the human race, the biggest decline slope in the global economy and mental trauma. A number of viruses have been discovered that may cause serious problems and to overcome this problem, early diagnosis of the viruses and understanding their infection pattern is a must. Early detection of viruses inside the host provides timely management in a strategic manner. Scientists have developed some effective and efficient methods to detect the viruses. In this review, we have explained a few types of diagnostic techniques: Biosensor based, immunological-based, and molecular-based diagnostic techniques that are prominent methodologies to identify and detect the course of infection related to the medical viruses. In biosensor-based diagnostic technique, an analytical device consisting of biological elements and physicochemical component gives a signal upon detection of viral antigen. In immunological-based diagnostic techniques, enzyme-linked antibodies are utilized to find the particular antiviral antibody or viral antigen in human specimens, and nucleic acid-based diagnostic techniques are based on the principle of amplification of the viral genome.

**Graphical abstract:**

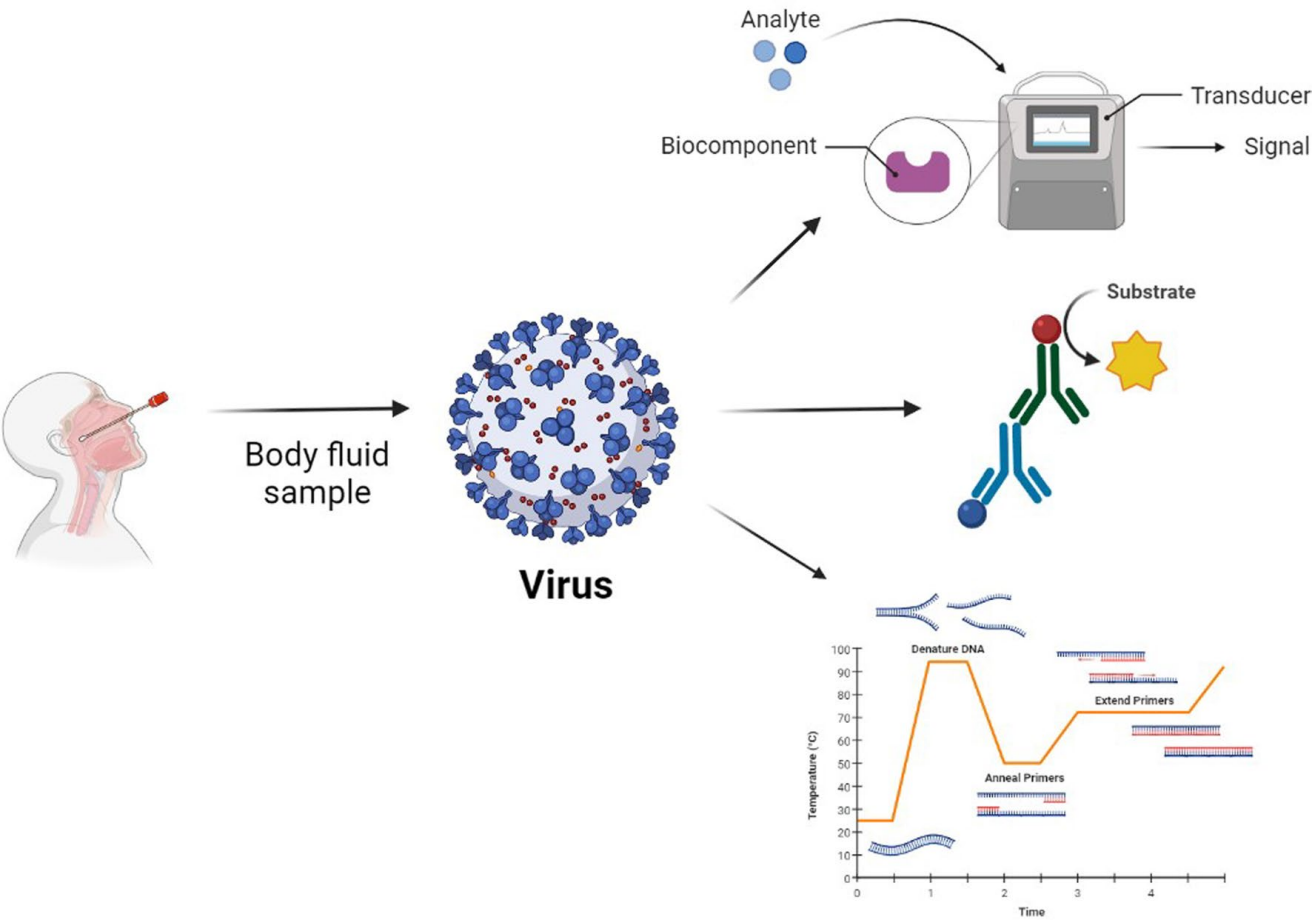

## Introduction

A virus is an infectious microorganism made up of a nucleic acid segment, which may contain any type of genetic material like deoxyribonucleic acid (DNA) or ribonucleic acid (RNA) coated inside a protein layer. A virus cannot multiply on its own; instead, it must infect cells and require host cell components to proliferate. Once within the host cells, viruses take control of/ hijack the biosynthetic machinery of the host to replicate their genetic material. Nowadays viruses are the major cause of human diseases. SARS-CoV-2, Human immunodeficiency virus (HIV), and hepatitis viruses are still responsible for millions of deaths globally. The human population is also facing significant issues as a result of the newly emerging viruses.

Since 2019, SARS-CoV-2 virus (COVID-19) had been the talk of the town and headed the news columns in all the newspapers.. The WHO labeled the COVID-19 outbreak a worldwide pandemic on March 11, 2020. It has become quite dangerous because of the continuous mutation with over more than 12,000 mutations been reported [1]. Omicron variant (B.1.1.529), which has been narrated as a matter of concern by The United States of America [2] is a deadly form ofCovid-19 and has caused more than 6 million fatalities around the globe [3].

Recently a viral breakdown, monkeypox has been observed in some countries. Monkeypox is a zoonotic disease that looks similar to smallpox and it is caused by orthopoxvirus. It was discovered first time in monkeys hence the name was coined, in 1958 [4] and the first human case was diagnosed in 1970 in a 9 months old baby [5]. At present, there are more than 45 thousand confirmed cases around the globe [6]. As a preventive measure, effective diagnostic methods are needed to identify these viral infections promptly and precisely. Early and precise diagnosis of viral diseases is crucial for the appropriate selection of the best treatment, reduction of treatment costs, and disease control. It also contributes to the development of disease prevention and treatment techniques, such as the creation of antiviral vaccines and novel therapeutic drugs [7].

Earlier, the proliferation of medical viruses were diagnosed in swine tissue culture [8], in animals [9] and through visual evaluation using electron microscopy [10]. Conventional diagnostic methods sometimes include laborious, costly, time-consuming, and unreliable procedures [11].

## Types of diagnostic techniques

### Biosensor based diagnostic techniques

A biosensor is a small but very sensitive and selective analytical device that consists of a biological element and a physicochemical component; it gives a signal upon detection of an analyte. The principle of the biosensor is based on the fact that the target analyte gets bind with the bioreceptor and this complex gets examined through reaction, adsorption, or some specific processes, then the transducer translates the chemical alteration that further gets detected by a detector [12]. Nucleic acids, enzymes, RNA, complementary DNA can be used as biorecognition elements for the bioreceptor [13].

We can categorize biosensors as electrochemical, piezoelectric, and optical. The electrochemical biosensor is a device that contains bioreceptor elements which may be in a form of biocatalysts (enzymes, tissues) or affinity sensors (antibody, nucleic acid) that gets reacted with our target analyte which may be antibody-antigen, proteins, etc. This whole biochemical reaction is observed by a transducer (Potentiometric, amperometric) and converts into electric signals and gets displayed on the detector [14]. Most recently this technique has been used to detect SARS-CoV-2 with the help of a Field-effect transistor (FET) based biosensor. Firstly the graphene sheets were rolled over the FET then 1-pyrene butyric acid N-hydroxysuccinimide ester (PBASE) coupling agent was used to adhere the anti-spike protein antibodies (SpAb) to the probe. A nasal swab of COVID-19 patients was used as a sample. Spike protein of viral antigen can be detected in phosphate buffer saline and another medium. The LOD of cultured was 2.42 to 102 copies/mL [15]. Surface imprinted polymer (SIPs) composites have been implemented in this technique to detect the Zika virus in 10% serum sample and the LOD was 2 × 10^−3^ PFU/mL (10–250 RNA copies/mL), an electric pulse-induced electrochemical biosensor has been used to detect the Influenza virus with the LOD of 3 × 10^−10^gmL^−1^ [16].

The second one is piezoelectric transducers, quartz crystal microbalance (QCM) biosensor is the most common type as it has a high specificity and portability. In this, the biomolecules will be immobilized on the surface of the oscillating crystal and this interaction will be sensed by QCM. Lower oscillation means a higher binding reaction hence mass [17]. For the detection of COVID-19, fabricated lithium niobate cantilevers are used where the SARS-CoV-2 spike proteins are immobilized and SARS-CoV-2 capture antibodies were added after this, with the help of tone burst signal excitation of the cantilever is done. A detector detects the SARS-CoV-2 capture antibodies with the help of an FFT signal. This technique is also applicable for the detection of the Human Papillomavirus (HPV) from human cervical specimens by optimizing the sensor with synthetic oligonucleotides [18].

The third one is the most common type of biosensor, the optical biosensor. It is very convenient because it enables the real-time and label-free detection of biological substances. These biosensors detect the change in optical behavior of the surface of the transducer when an analyte and bioreceptor form a complex. Some optical transducers are used in this which are Interferometer, Surface Plasmon resonance (SPR) biosensor, Surface-enhanced Raman scattering (SERS), fluorophores, or chromophores are also used to detect the binding events [19].

Optical biosensors can be used to detect viral antigens inside the sample like it has been used for the detection of CoVID-19 in the biological sample where SPR is used to detect the RNA-dependent RNA polymerase (RdRp) which is very similar to the SARS-CoVID, in this 200 μL of analyte solution is used which roughly contains 2.26 × 107 copies of the RdRp-COVID sequence. The biosensor which was used was coated with a gold surface that contains the cDNA, when some heat was given it makes immobilized the RdRp [18]. Recently Petrovszki et al. used the optical biosensor to find out whether the S1 subunit of SARS-CoV-2 spike protein can cross the Blood–Brain barrier or not, a study reported that spike protein S1 subunit could cross the Blood–Brain barrier hence this model can be used for further studies [20].

### Immunological diagnostic techniques

As we all know that human body is capable to demolish foreign invaders which are in the form of pathogens, viruses, bacteria, etc. with the help of antibodies against the particular antigen. This process is controlled by humoral immunity which is a part of the immune system. With the help of this antigen–antibody complex concept, scientists have developed immunological-based diagnostic techniques. Enzyme-linked immunosorbent assay, western blotting, and immunofluorescence assay are some examples of Immunological based diagnostic technique [21].

#### Enzyme-linked immunosorbent assay (ELISA)

**Fig. 1:**
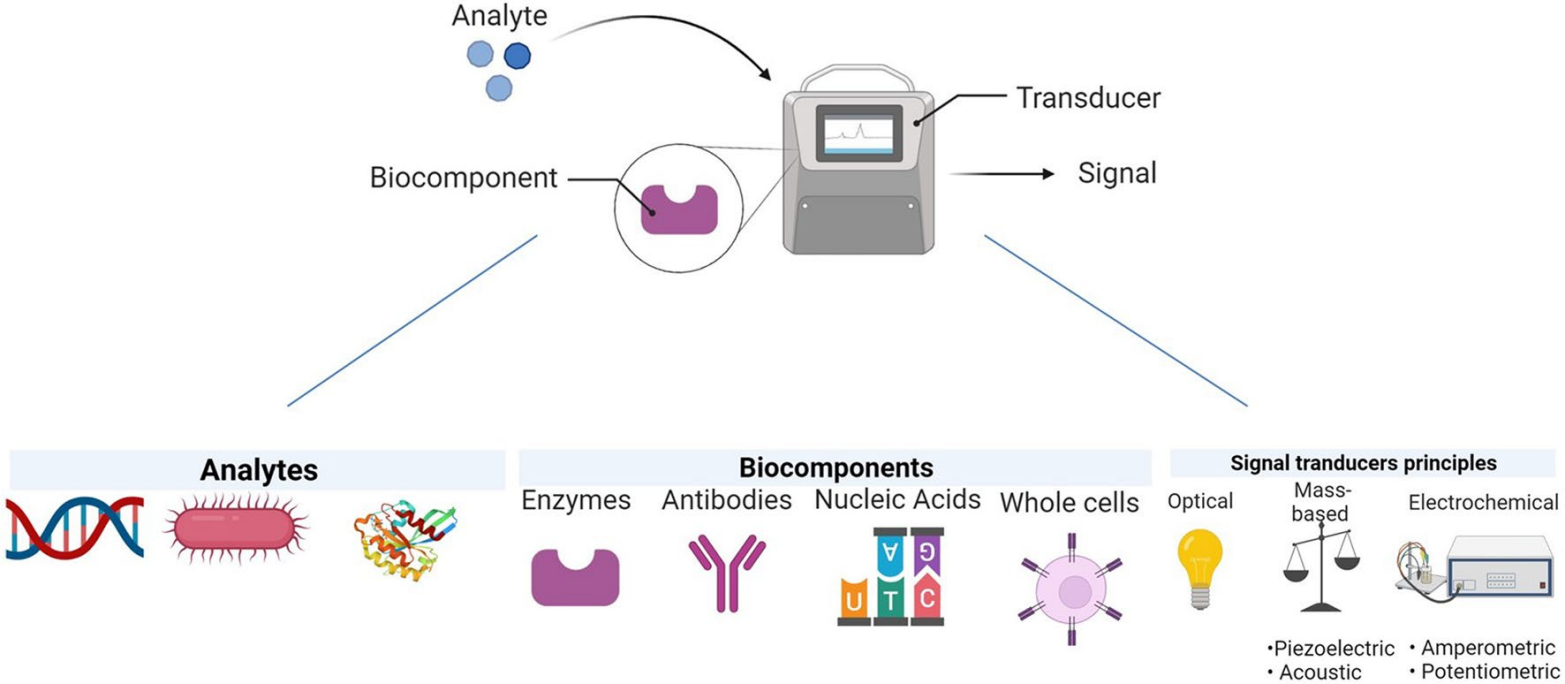
Diagrammatic presentation of Biosensor. The analyte which could be anything right from enzymes, antibodies, nucleic acids, or even whole cells, when binds with the biocomponent, gets detected by the transducer on the basis of an optical, mass-based, electrochemical mechanism and sends a signal that could be further analyzed to diagnose the presence of a viral particle in the body

The Enzyme-linked immunosorbent assay (ELISA) is the biochemical assay that is commonly used to find viral infection inside specimens. Where the enzyme-linked antibodies are utilized to find the particular antiviral antibody or viral antigen in human specimens. The principle is that the specific antibody binds with the target antigen and the quantity of binding gets detected with the help of a colorless chromogenic substrate which results in the creation of a colorful product. No color is created in the clinical specimen when there is no antigen or antibody present and the intensity of the color is equal to the amount of antigen–antibody formed [22]. A spectrophotometer is used for the detection of color shift. Scientists have developed several types of ELISA, among them there are two important types of ELISA (Fig. 1).

**Fig. 2:**
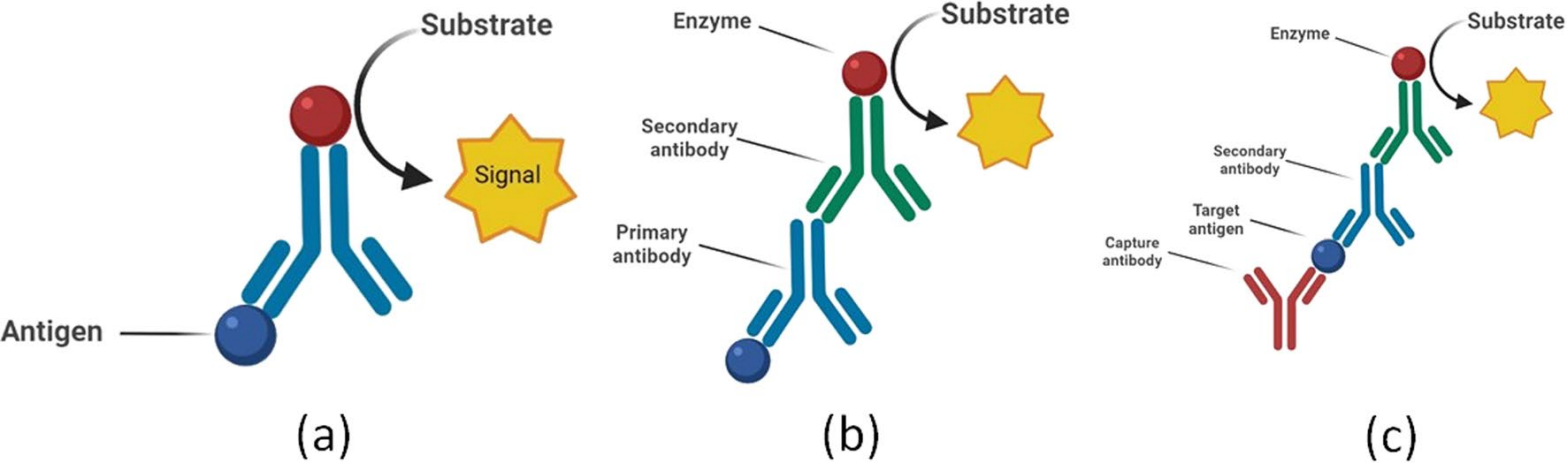
**a** Direct ELISA, **b** Indirect ELISA, **c** Sandwich ELISA

1. Sandwich ELISA is also known as antigen-capture ELISA.
2. Indirect ELISA is also known as antibody-capture ELISA.
3. As we can see in Fig. 2 in sandwich ELISA there are two antibodies; one is a capture antibody and one is a detection antibody. The antigen which we want to find out is bound between the capture antibody and detection antibody hence this method is known as a sandwich antibody. The capture antibody adheres to the surface and the detection antibody which is conjugated with any enzyme or fluorophore label is used in the last just because of quantitation [23]
4. Indirect ELISA is a two-step process of binding primary antibody and labeled secondary antibody. Firstly incubation of the primary antibody with antigen and then the incubation with secondary antibody. This, however, may result in nonspecific signals due to cross-reactions caused by the secondary antibody [24].

ELISA is simple to use, sensitive, and specific, and it produces data quickly. The test was created as a result, and it is now widely used for the identification and serosurveillance of human viral infections. ELISA has been widely used recently for the detection of SARS-CoV-2 IgM or IgG antibody inside human plasma samples. ELISA is also used to detect Ebola virus, HSV-2 virus, SARS-CoV, Hepatitis virus, Monkeypox virus [25].

#### Western blotting analysis

For the past four decades, Western blotting has been a vital analytical tool in cell and molecular biology and it is also known as an immunoblotting assay. For the detection of viral proteins, Proteins are separated in SDS-PAGE, then electrotransferred to a nitrocellulose membrane, and then incubated with antibodies conjugated enzymes specific for viral proteins [26]. If the viral proteins get bound with antibodies conjugated enzymes chromogenic substrate will be added which leads to the formation of a colored band near the viral antigens. One can isolate a particular protein from a complicated mixture of proteins using a western blot. It may be utilized as a diagnostic tool as well as to confirm proteins of interest in exploratory proteome research to uncover various disease processes. Western blot is widely used in proteome research due to its simplicity, affordability, and accessibility [27]. Western blotting analysis can be used to detect several viral infections Xing et al. [28] developed a chimeric antigen to detect the antibodies against the rubella virus, Wei T et al. [29] also developed the antibodies for the detection of coronavirus***,*** human immunodeficiency virus with the specificity of nearly 100% [30]*.*

#### Immunofluorescence assay

The immunofluorescence assay (IFA) is also known as immunostaining and is a classic virologic approach for detecting antibodies based on their unique capacity to react with viral antigens produced in infected cells; bound antibodies are seen by incubating with fluorescently labeled antihuman antibodies. Prefixes like cyto and histo are used during the description of the antigenic site which is inside the cell or tissue and on the other hand suffixes like fluorescence and chemistry is used to state whether fluorochromes are used for the labeling of antibody or enzymes are used. Conjugated antibodies provide either enzymatic or fluorescent signals, which are then connected to bright-field or fluorescence microscopy, respectively [31].

Other than the use of enzymes and fluorochromes this assay is subdivided into two categories direct and indirect. In the indirect technique, a labeled secondary antibody detects a non-conjugated primary antibody that is intended to connect to its antigenic affinity site, as opposed to direct immunostaining, which only applies when a primary antibody directly binds to a label (conjugate antibody). The direct method has less sensitivity as compared to the indirect method nevertheless it is preferable by a researcher to the indirect method [32].

In most cases, IFA is found to be more specific and sensitive as compared to traditional cultural techniques [32].

### Molecular diagnostic techniques

There is a huge dependency of diagnostic virology on molecular diagnostic techniques as it is fast, highly sensitive, and has high specificity (Table 1). Nucleic acid-based molecular detection is the most famous one [33].

**Table 1:** Diagnostic techniques with their sensitivity, strength and weakness

Technique	Sensitivity	Strength	Weakness	References
Biosensors	Sensitive	High specificity	Expensive	[14]
PCR	Sensitive	Wide variety of pathogens can be detected with various modifications More sensitive than antigen and antibody detection	Susceptible to specimen contamination which leads to false results Use time and labor Proper instrumentation required	[34]
RT-PCR	Moderately sensitive	Capable of detecting multiplexes	Chances of contamination is high RNA is difficult to handle	[35]
Real-time PCR	Sensitive	Less cross contamination risk Time cosumption Quantitative detection	Expensive equipment and probe required Designing of TaqMan probe needs full knowledge of target nuclei	[36]
TMA	Moderately sensitive	Highly specific Rapid Thermal cycle not required	RNA is not easy to handle Multiple enzymes needed	[37]
DNA micro arrays	Sensitive	Can detect several pathogens simultaneously Used for the detection of both antibody or nucleic acid	Highly expensive for majority of patients Usually needs amplification of nucleic acids before analysis	[38]

#### Nucleic acid-based amplification techniques

This method is based on the principle of amplification of the viral genome. It is very sensitive, and specific and provides a quick diagnosis. We can detect different viruses at the same time. Nucleic acid-based amplification methods are widely used for the detection of those viruses which are very fragile, and difficult to cultivate. With the help of nucleic acid amplification, we can detect HIV, hepatitis C virus, ZIKV, coronavirus, and monkeypox virus [39].

##### Polymerase chain reaction (PCR)

PCR has been invented by K. B. Mullis and F. A. Mullis in the year 1985 and since then commonly used for nucleic acid amplification [40].

PCR enables the replication of millions of identical DNA copies from a little quantity of the pathogen genome in a clinical sample [41].

In theory, firstly the target DNA was extracted, followed by heating and denaturation. Specific oligonucleotide primers are annealed to the DNA at a lower temperature after that the DNA polymerase enzyme replicated the template strand known as the extension phase. This cycle is frequently repeated by 30–40 times, producing millions of identical DNA copies. Following the PCR process, the amplified result can be found using a variety of methods, including colorimetric assays, sequencing, and gel electrophoresis [42].

Since the discovery of PCR, it has been used to identify viral infections with a sensitivity of more than 77% and a clinical specificity of more than 85% [34].

PCR is used to diagnose viruses, bacteria, parasites, and fungi. Infections such as poxviruses, enteroviruses, herpes viruses (HSV, ZSV, CMV), coronavirus, and monkeypox as well as a general or specific bacterial screening of Neisseria meningitides, streptococcus pneumonia, bordetella pertussis, and borrelia spp. are frequently detected using qualitative PCR [43]. In quantitative PCR the difference of copy numbers of nucleic acid in a sample with control of known copy number is compared, this method is used to detect viral infections like HIV, CMV, and EBV [44].

Some clinical laboratories still employ traditional PCR, although more recent variations of the technology are quickly replacing it.

The PCR method is quite flexible. A variety of PCR variations have been created, although reverse transcription-PCR and real-time PCR are the most significant variations. The former was developed to amplify RNA targets and the latter was developed to quantify DNA in real-time throughout the PCR reaction [40].

##### Reverse transcription-PCR (RT-PCR)

The RNA targets are amplified using RT-PCR. This technique involves employing reverse transcriptase (RT) to convert viral RNA targets into complementary DNA (cDNA), which is subsequently amplified using conventional PCR [35]. Since its introduction, RT-PCR has been utilized to diagnose human infections caused by RNA viruses. The sensitivity of RT-PCR is ranging from 73 to 100% and the specificity is almost 99% to 100%. These findings suggest that RT-PCR is a good approach for detecting RNA virus infections in humans. However, due to the method's high cost and labor-intensive technique, it is no longer frequently employed with clinical specimens [34].

##### Real-time PCR

Real-time PCR systems perform viral nucleic acid amplification and detection simultaneously. The amplified product can be detected by the amount of fluorescence emitted by the specimen. The Fluorescence emitted by the specimen is monitored by some special thermal cycler. The computer, which is linked to the thermal cycler through suitable software, captures the data and generates an amplification plot at the end of each reaction cycle [45].

The technologies of SYBR green, TaqMan, and molecular beacon may all be utilized to recognize and quantify amplification products. The SYBR green dye attaches to the minor groove of the double-stranded DNA (dsDNA) product when exposed to enough light, and this increased fluorescence is proportional to the accumulated dsDNA product [46].

The TaqMan probe is a DNA oligonucleotide with a quencher (3′ bases) and reporter (5′ bases) fluorescent dyes attached to either end.

TaqMan probes have a specific internal region of a PCR product that they are made to bind to. During the annealing stage of the PCR, the TaqMan probe and the primer clung to the template strand. Taq DNA polymerase uses its 5′-3′ exonuclease activity to cleave the probe after stretching the primer. When the probe cleaves, the fluorescent dye is released, resulting in fluorescence emission. (Fig. 4) A relationship exists between the amount of fluorescence and the amount of PCR product [47].

A molecular beacon is a small DNA molecule with a quencher and fluorescent dye on opposite ends. The 3′ and 5′ end sequences complement one another. The internal component of the molecule is designed to complement the target sequence in a PCR product [48].

Fluorescence emission happens when a molecular beacon combines with the target sequence and separates the fluorophore and quencher. The amount of PCR product is directly correlated with the amount of fluorescence [49]. Real-time PCR is very famous among laboratories around the globe for the finding and quantification of medical viruses in any given sample because this technique is very sensitive and specific [50]. When compared to conventional quick culture, Ross et al. [45] 's real-time PCR method had an overall sensitivity of 100% and specificity of 99.9% for the detection of cytomegalovirus (CMV) in liquid saliva.

When compared to serologic tests, the approach was also used to diagnose primary Epstein–Barr virus (EBV) infection, with a sensitivity of 95.7% and specificity of 100%. Realtime PCR was also used to assess the viral load in patients with herpes simplex encephalitis [40]. The measurement of viral loads in patient samples is essential because it offers prognostic and predictive data. Patients in this trial were shown to need longer courses of acyclovir medication and to have worse clinical outcomes than those with lower virus loads in their cerebrospinal fluid (CSF) [51]. The test may also be used to identify many viruses in a multiplexed manner. TaqMan probe and molecular beacon both play critical roles in the multiplex detection of various viruses in a single PCR reaction. Distinct fluorescent dyes are used to mark different probes/beacons in multiplexing experiments [52]. Multiplex tests for the detection of human adenovirus B, C, and E in throat swab samples have demonstrated that real-time PCR has a 99.6% specificity and 100% sensitivity [53]. Using CMV, EBV, HSV-1 and HSV-2, JEV, and varicella-zoster virus as examples, For the identification of neurotropic viruses in CSF, Ramamurthy and colleagues [54] compared multiplex real-time PCR to multiplex conventional PCR (VZV).

Real-time PCR revealed viral infections in 88 of the 147 CSF samples taken from individuals with neurological diseases, but traditional PCR could only identify the viruses in six of the samples, indicating that real-time PCR had better sensitivity than conventional PCR [45].

Scientists have developed a triplex quantitative real-time PCR test for the detection of human adenovirus (hAdv) serotypes 2, 3, and 7, this assay is very fast and efficient and the sensitivity in the form of the limit of detection; LOD was found to be 102 DNA copies per reaction and no cross-reactivity with any other respiratory viral infection were found [55].

Real-time PCR may be used with traditional reverse transcription PCR (RT-PCR) to create reverse transcription quantitative real-time PCR by adding the reverse transcription step (RT-qPCR). The advantages of RT-qPCR over traditional RT-PCR include reduced contamination, the ability to quantify the amplicons, and a short assay time because no post-PCR processing is required [35].

The identification and quantification of various RNA viruses in clinical specimens, such as ZIKV, Ebola, coronavirus, HCV, SARS-CoV-2, respiratory syncytial virus (RSV), dengue virus, HIV-1, and influenza A virus, are therefore often accomplished by RT-qPCR. Recently, Corman et al. [32] developed a one-step multiplexed RT-qPCR for the detection of the African swine fever virus (ASFV). The assay can detect the ASFV, classical swine fever virus (CSFV), and atypical porcine pestivirus (APPV) simultaneously with the LOD of the assay 2.52 × 101 copies/μL. The intra- and interassay coefficients of variation (CVs), as determined by a repeatability test using conventional recombinant plasmids, were less than 2% [36].

##### Transcription-based amplification methods

Two methods come under the transcription-based amplification method which is transcription-mediated amplification (TMA) and nucleic acid sequence-based amplification (NASBA). These two techniques are very similar to one another and also know as the isothermal amplification method. The entire amplification process takes place at a temperature of 41 °C [56]. In both procedures, RT converts the viral RNA target into cDNA then RNA polymerase produces multiple copies of the viral RNA output. Among these two only TMA uses two enzymes (RT and RNA polymerase).

The whole amplification reaction is carried out at the predetermined temperature of 41 °C. Avian myeloblastosis virus (AMV) reverse transcriptase (RT), RNase H, and T7 DNA-dependent RNA polymerase are the three enzymes involved in this homogenous isothermal process (DdRp). The method is especially well suited for RNA analytes like mRNA, rRNA, or genomic RNA since RT is incorporated into the amplification process [57].

**Fig. 3:**
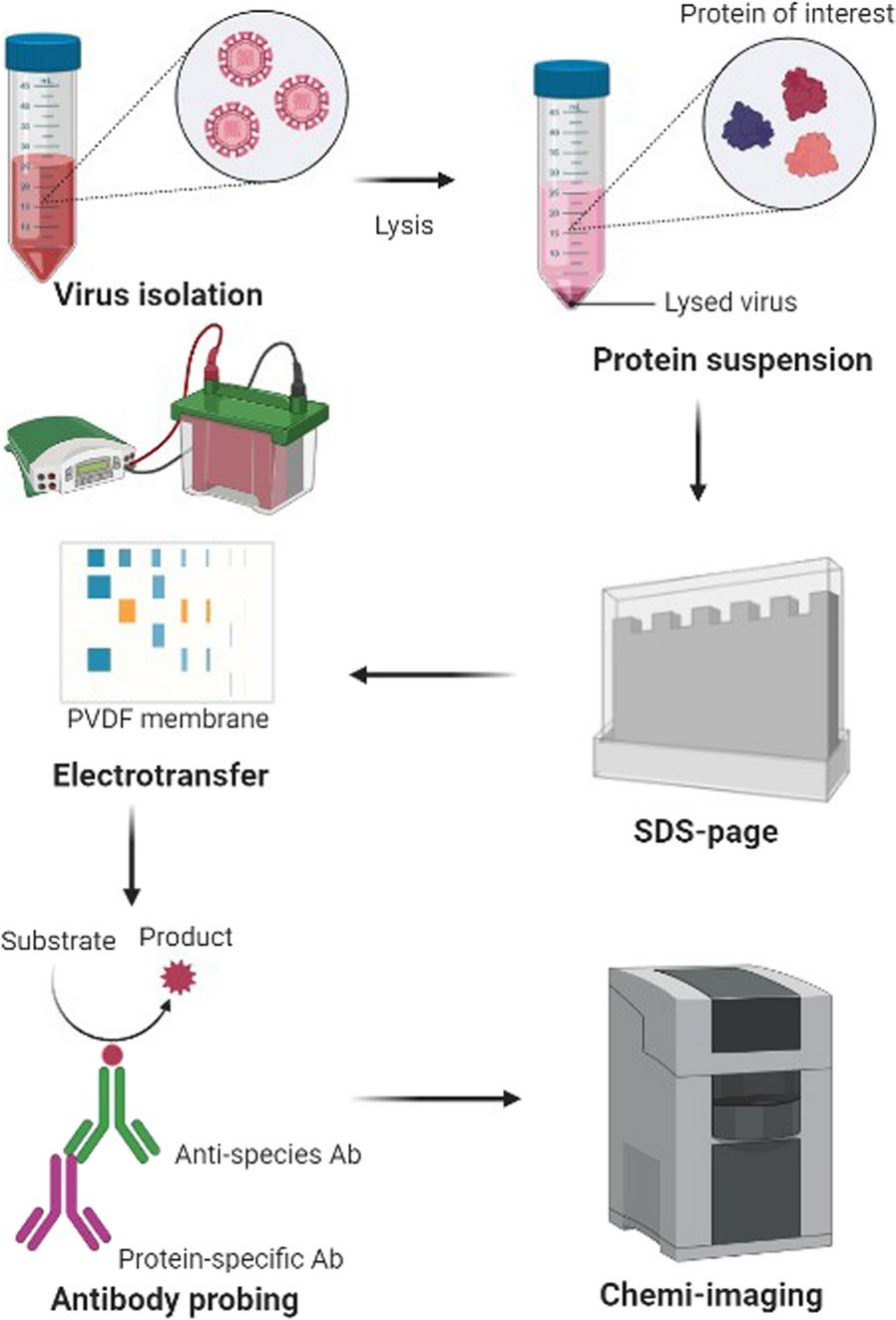
Step wise representation of western blotting technique. It starts with the isolation of virus then with the help of lysis buffer protein of interest has been separated then run SDS-page after that the separated protein transferred into PVDF membrane then the antibody probing has been done and results has been collected by chemi-imaging

Three enzymes and two primers work together in the NASBA procedure to exponentially amplify a target viral RNA, as shown in Fig. 3. Primer 1 (P1) has a T7 RNA polymerase promotor region at its 5′ ends and a complementary sequence to a target viral RNA sequence at its 3′ ends [58].

Primer 2 (P2) contains a sequence that is complementary to the cDNA strand. The viral RNA is converted into a cDNA copy by reverse transcription (RT) utilizing P1 to start the amplification process. RNase H breaks down RNA–DNA hybrid molecules that contain viral RNA. RTsynthesizes dsDNA molecules utilizing P2 and the released DNA strand. Last but not least, T7 RNA polymerase creates several copies of viral RNA using dsDNA molecules as templates [59].

**Fig. 4:**
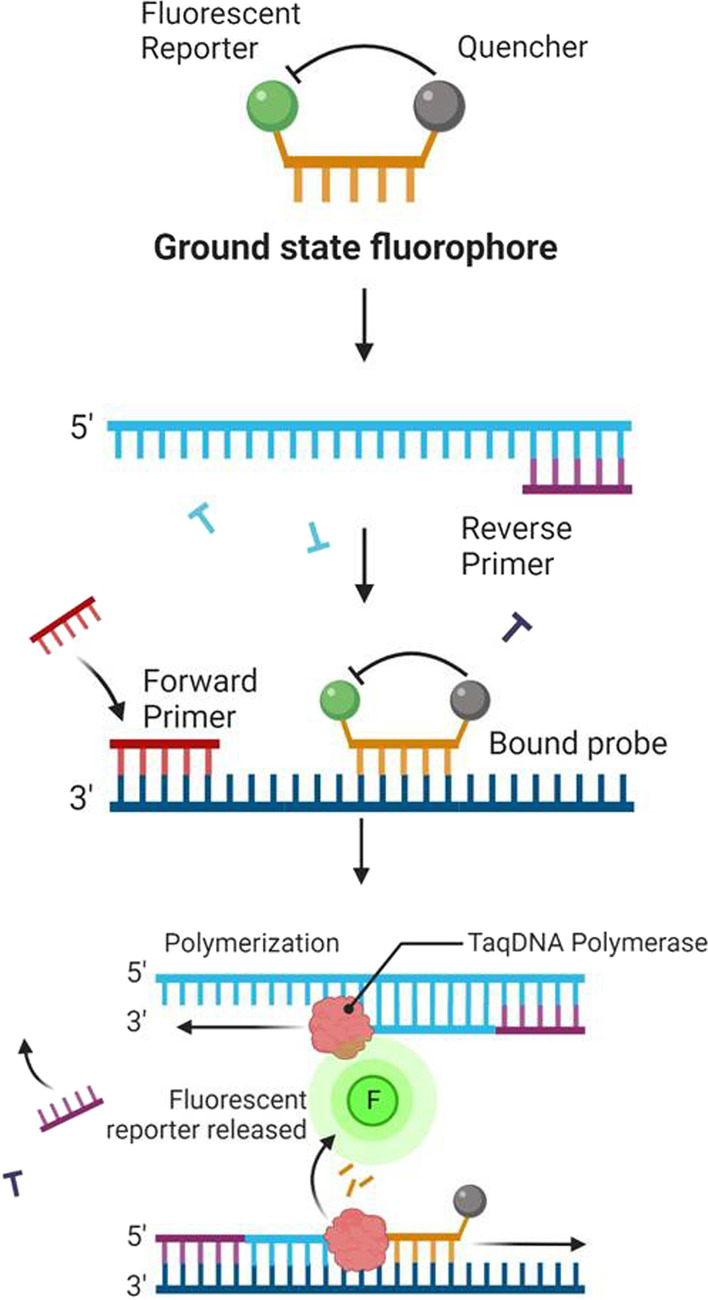
diagrammatic representation of quencher and TaqDNA polymerase in Real-time PCR

An accumulation of many viral RNA copies and ds DNA molecules happens as a result of several repetitions of the cycle mentioned above. At the end of the test, gel electrophoresis can be used to identify the amplified product, or a molecular beacon can be used to detect it in real-time. A molecular beacon can also be used in the detection of NASBA products (Fig. 4). As we have discussed earlier molecular beacons are DNA oligonucleotides that are labeled with a fluorophore at the 5' end and a quencher at the 3' end [60].

The sequence at the very 3' end is complementary to the sequence at the very 5' end, and a hairpin stem is created in such a way that the quencher absorbs the fluorophore's emitted light. The hairpin loop sequence is complementary to the amplicon's target sequence. Because the loop sequence binds to the target, the hairpin stem opens and the quencher is separated from the fluorophore [49].

**Fig. 5:**
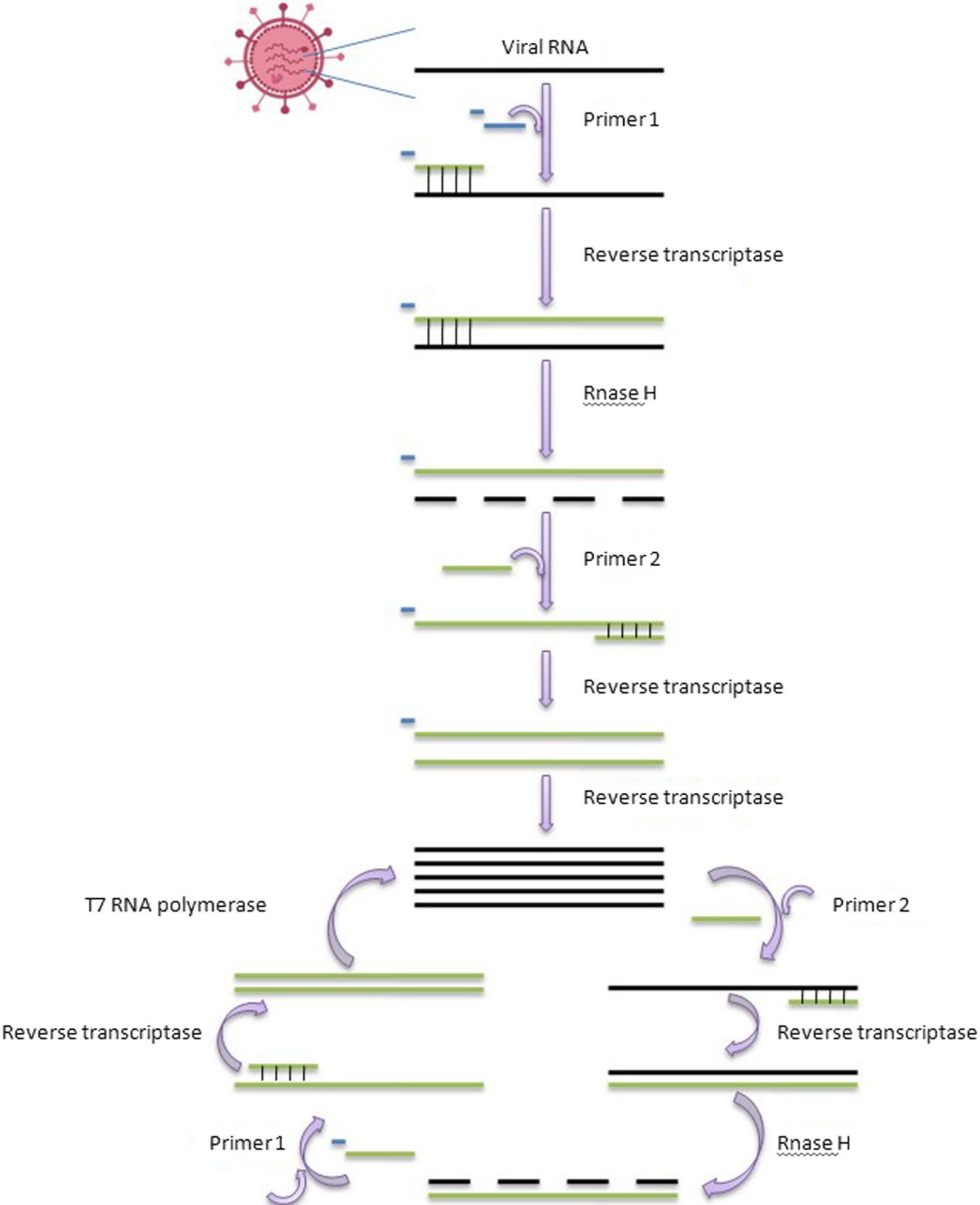
Schematic view of principle of NASBA

NASBA has some advantages the amplification of the nucleic acid sequences of more than 10 9 copies can be done in just 90 min by using three enzymes. In NASBA there is no need for a thermal cycler because the whole process has been carried out at 41 °C (Fig. 5). A main advantage of NASBA is the production of single-stranded RNA amplicons which can be used directly in another round of process or can be used as a probe for detection without denaturation or the separation of the strand [61]. In NASBA we can detect the human mRNA sequences without any DNA contamination. Some Applications of NASBA are:-

1. For the detection of Infectious agents.
2. For the detection of Blood borne pathogens like Human immunodeficiency virus genomic RNA, Hepatitis C virus genomic RNA, Enterovirus genomic RNA [56, 62].
3. For the detection of Respiratory pathogens like Mycoplasma pneumonia, Mycobacterium tuberculosis [63].

##### Loop-mediated isothermal amplification (LAMP)

The term "Loop-Mediated isothermal amplification" (LAMP) refers to another isothermal nucleic acid amplification method that is frequently utilized for the rapid, simple, accurate, and cost-effective detection of both DNA and RNA viruses in human specimens. This technique is particularly well-liked in the field of diagnostic virology and was initially introduced by Notomi et al. [64].

In this method, the stem-loop structure generated at the 3'-terminus is employed as a template for self-elongation from the loop region, and additional primers are subsequently bound to the loop region and extended there. This reaction uses a pair of inner and outer primers. Each inner primer has a sequence at the 3'-terminus that is complementary to one chain of the amplification area and identical to the inner region of the same chain at the 5'-terminus [41].

DNA polymerase-mediated strand-displacement synthesis, which employs the abovementioned stem-loop sections as a step, carries out the elongation activities sequentially. The fundamental goal of this process is to produce a sizable number of DNA amplification products with an alternating, repeating structure and a mutually compatible sequence [65].

The fast, sequential progression of the LAMP amplification response leads to its high amplification efficiency. Additionally, little amounts of a gene can be amplified quickly (Nagamine et al., 2002) Specific loop primer designs can cut amplification time in half or one-third of that of the original LAMP method. The addition of the RT in this LAMP process (RT-LAMP) will permit the amplification of the RNA target [66]. The LAMP reaction is carried out at a constant temperature of 60–65 °C, eliminating the need for expensive specialist equipment. The approach requires just a low-cost heating block or water bath, making it ideal for usage in small laboratory settings [67].

There are several ways to determine the amplified product, including using a turbidimeter to measure the turbidity brought on by precipitated magnesium pyrophosphate in real-time, viewing the precipitation of magnesium pyrophosphate after the reaction is finished, adding intercalating fluorescent dye to the final reaction mixture and using an agarose gel electrophoresis to see the bands of different diameters to detect fluorescence under ultraviolet or natural light [68].

With no cross-reactions with other chosen viruses, LAMP has been employed for the quick detection of a few DNA viruses in human specimens, including HSV-1, hAdV40, and hAdV41. According to several pieces of research, RT-LAMP is more sensitive than NASBA and conventional RT-PCR [69].

Additionally, this technique has been altered to conscientiously identify dengue virus, HIV-1, and ZIKV in clinical samples. For the detection of SARS-CoV-2 in respiratory species, certain commercial RT-LAMP kits are available [70].

It is based on a system that combines clustered regularly interspaced short palindromic repeats-CRISPR-associated protein 12a (CRISPR-Cas12a) cleavage assay with reverse transcription loop-mediated isothermal amplification (RT-LAMP), which enables the identification of particular HCV nucleic acid sequences.

##### DNA microarrays

DNA microarrays are electrical engineering technologies that are used to evaluate the expression of medical viruses [38]. Fluorescently marked viral nucleic acids are used in DNA microarray diagnostics to screen an array of oligonucleotide probes that are typically placed on silicon, glass, or plastic solid surfaces. Larger DNA/RNA fragments, amplicons, or synthesized oligonucleotides can all be used as probes [71]. The probes which are used here are particular to the target virus's genome. Fluorescence-based detection is used to identify and quantify the outcomes of the hybridization between immobilized probes and fluorescently-labeled target sequences [72]. If we compare this test with other diagnostic techniques we will get 80% to 90% overall specificity, but it will go up to 99% when we talk about specific rhinovirus, influenza A virus, and 93% for meningitis virus, and encephalitis virus [71]. This test shows LOD of 10 GE/ reaction for almost every viral infection and with nill cross reactivity. We can use DNA microarrays for the specific detection of viruses that are present inside the gastrointestinal tract, viruses that are transmitted by arthropods, and some other common viruses with high specificity [73].

During the 2002 SARS outbreak in China, DNA microarray assisted in the identification of a novel member of the coronavirus family [74]. However, the method does have certain drawbacks, such as being too costly to be employed for routine clinical diagnosis and labor- and time-intensive (the hybridization process may take hours to days to complete). The sensitivity of the assay may be impacted by non-specific hybridization between test materials and immobilized probes. Additionally, nearly all of the genetic information about the target virus's composition is needed for constructing specialized probes. Only viral pathogens with target probes on the array are detected by the test [75].

## Future challenges and opportunities

This article focuses on the various aspects of diagnosis issues in viral diseases and hence also highlights the different instrumentation techniques and devices used or should be used in detecting the virus and studying the etiology of diseases caused by the medical viruses. This article brings together the various aspects of the logical analysis in virology. Its innovation in its own term that all the types of molecular, immunological, genetic, pharmacological and pharmaceutical implications of medical viruses diagnosis have been bought under this one umbrella articl. For the quick identification of SARS-CoV-2, the FDA has approved 235 molecular tests, while the EMA (European Medicine Agency) has approved 192 PCR-based methods. The most accurate molecular diagnostics for a COVID-19 diagnosis are those based on RT-PCR. In South Korea, if a sample is conveyed in UTM (universal transport medium) or molecular water, SARS-CoV-2 may be detected directly by rRT-PCR without the requirement for RNA extraction [76]. Additionally, a number of businesses have created customized RT-PCR assays that have been given emergency use authorisation by the United States FDA, such as Cobas® Liat® (Roche Molecular 145 Systems, USA), Xpert® Xpress SARS-CoV-2 (Cepheid, USA), and ID NOWTM (Abbott, USA). For the purpose of detecting SARS-CoV-2 in India, the Sree Chitra Tirunal Institute for Medical Sciences and Technology Thiruvananthapuram has created the Chitra GeneLAMP-N diagnostic test kit. One of the first confirming diagnostic tests for the SARS-CoV-2 N-gene in the world, this kit yields results in two hours. Moreover, it is necessary to improve the RT-PCR methodology to tackle the problem of less than perfect sensitivity [77].

## Conclusion

The use of nucleic acid-based diagnostic techniques in diagnostic virology has greatly improved the diagnosis of human viral infections. To diagnose and manage medical viruses, nucleic acid-based diagnostic techniques are extremely sensitive and specific. Viral RNA or DNA is identified using molecular diagnostic techniques to identify viral infections. These methods can therefore identify sick people before an immune response is developed against the relevant pathogen. Patients who are young, old, or immunosuppressed should pay extra attention to this. However, because of their expensive price, complicated equipment, and need for professional skills, they are out of the reach of countries with few resources. Worldwide, immunoassays are crucial for the diagnosis and monitoring of viral infections. Even immune-based diagnostic techniques are very easy to perform and pretty inexpensive as compared to molecular-based diagnostic methods, they are not widely available and used in under-developing countries. Consequently, researchers are trying to elaborate and develop new techniques which will be more specific, less expensive, and easy to use.

## Data Availability

Data and material can be made available for use by the journal.
